# Agreement between medical students’ peer assessments and faculty assessments in advanced resuscitation skills examinations in South Korea

**DOI:** 10.3352/jeehp.2021.18.4

**Published:** 2021-03-25

**Authors:** Jinwoo Jeong, Song Yi Park, Kyung Hoon Sun

**Affiliations:** 1 Department of Emergency Medicine, Dong-A University College of Medicine, Busan, Korea; 2 Department of Medical Education, Dong-A University College of Medicine, Busan, Korea; 3 Department of Emergency Medicine, Chosun University College of Medicine, Gwangju, Korea; Hallym University, Korea

**Keywords:** Clinical clerkship, Emergency medicine, Intratracheal intubation, Medical students, Peer review

## Abstract

**Purpose:**

In medical education, peer assessment is considered to be an effective learning strategy. Although several studies have examined agreement between peer and faculty assessments regarding basic life support (BLS), few studies have done so for advanced resuscitation skills (ARS) such as intubation and defibrillation. Therefore, this study aimed to determine the degree of agreement between medical students’ and faculty assessments of ARS examinations.

**Methods:**

This retrospective explorative study was conducted during the emergency medicine (EM) clinical clerkship of fourth-year medical students from April to July 2020. A faculty assessor (FA) and a peer assessor (PA) assessed each examinee’s resuscitation skills (including BLS, intubation, and defibrillation) using a checklist that consisted of 20 binary items (performed or not performed) and 1 global proficiency rating using a 5-point Likert scale. The prior examinee assessed the next examinee after feedback and training as a PA. All 54 students participated in peer assessment. The assessments of 44 FA/PA pairs were analyzed using the intraclass correlation coefficient (ICC) and Gwet’s first-order agreement coefficient.

**Results:**

The PA scores were higher than the FA scores (mean±standard deviation, 20.2±2.5 [FA] vs. 22.3±2.4 [PA]; P<0.001). The agreement was poor to moderate for the overall checklist (ICC, 0.55; 95% confidence interval [CI], 0.31 to 0.73; P<0.01), BLS (ICC, 0.19; 95% CI, -0.11 to 0.46; P<0.10), intubation (ICC, 0.51; 95% CI, 0.26 to 0.70; P<0.01), and defibrillation (ICC, 0.49; 95% CI, 0.23 to 0.68; P<0.01).

**Conclusion:**

Senior medical students showed unreliable agreement in ARS assessments compared to faculty assessments. If a peer assessment is planned in skills education, comprehensive preparation and sufficient assessor training should be provided in advance.

## Introduction

### Background/rationale

Traditionally, peer assessment in medical training has been used to obtain quantitative information on a trainee’s performance, with examples including peer ranking and peer rating [1]. In recent years, peer assessment has been considered to be an effective learning strategy [2]. In a study of objective structured clinical examinations (OSCEs), peer assessment was found to be beneficial for learners, who received high-quality feedback and experienced stress reduction, and for peer assessors (PAs) in terms of improved teaching and clinical skills [3]. Basic life support (BLS) skills are a key element of the undergraduate curriculum that medical students should acquire. Peer-led BLS training for medical students provided high-quality education that was as effective as professional training [4]. In studies of peer assessment in BLS training, senior medical students were able to make reliable assessments of their peers’ performance [5]. The interrater reliability between professional faculty assessors (FAs) and PAs has likewise been shown to be good [6]. However, quite a few studies of peer assessment in skills training were limited to BLS. Few studies have been conducted on peer assessment of advanced resuscitation skills, such as tracheal intubation and manual defibrillation, among medical students. To introduce peer assessment for educational purposes, its reliability and validity should be established, but studies have not provided sufficient psychometric data [7].

### Objectives

The objective of this study was to examine the agreement level between medical students’ peer assessments and a professional faculty member’s assessments in an advanced resuscitation skills examination. Thus, the research question of this study was as follows: How reliable is the peer assessment of medical students in an advanced resuscitation skills examination compared to that of professional faculty?

## Methods

### Ethics statement

This study was approved by the institutional review board of Dong-A University (IRB approval no., 2-1040709-AB-N-01-202101-HR-003-02). The requirement to obtain informed consent was waived because participants took part in the activity as part of their educational curriculum.

### Study design

This was a retrospective exploratory study aiming to determine the interrater reliability of peer assessment.

### Participants

In total, 54 fourth-year medical students who attended the emergency medicine (EM) clinical clerkship course were included in this study as PAs. They were divided into 8 groups (6–7 students per group) and participated in the course every 2 weeks from April to July 2020. A professional faculty member who was a qualified advanced life support instructor and emergency physician participated as an FA.

### Variables

The measurement variables were the BLS skills, tracheal intubation skills, and manual defibrillation skills assessed by peers and faculty.

### Setting

#### Resuscitation skills examination during the EM clinical clerkship

Students had already learned each resuscitation skill before the clerkship began. In the third quarter of the second and third years of medical school, they learned how to perform each skill and practiced the skill using manikins. When the clerkship began, a faculty member provided a 30-minute teaching session on how to perform each skill, which the students then practiced themselves for 2 weeks.

**Fig. 1.**
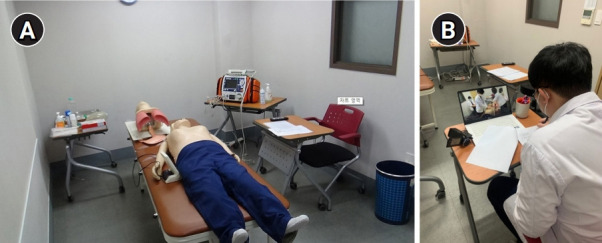
The station of the resuscitation skills examination. (A) The station configuration used a Recording Resusci Anne (Laerdal Medical, Stavanger, Norway) for basic life support, a defibrillation trainer (CU Medical Systems, Wonju, Korea) for manual defibrillation, and an airway trainer (Laerdal Medical) for tracheal intubation. (B) The last peer assessor assessing the videotaped peer’s skills performance.

On the last day of the 2-week course, a group of students participated in the advanced resuscitation skills examination. It was conducted as a formative assessment of the scheduled curriculum, and the topics evaluated were BLS, tracheal intubation, and manual defibrillation as 1 station of the OSCE (Fig. 1A). A faculty member evaluated and gave feedback on the students’ performance of resuscitation skills one by one. Another professional faculty member participated as an assistant and assessor trainer. The details of the station setup and the assessment process are presented in the authors’ previous report [8].

#### Development of a scenario and measurement checklist for the skills examination

The scenario and instructions for students were developed by 2 professors and were as follows. A 57-year-old man collapsed in the office and came to the emergency center via emergency medical services. The student is expected to evaluate the patient’s status and perform first aid. The time allocation was 12 minutes. This instructions and allocation was posted on the door outside the station. We used a Recording Resusci Anne (Laerdal Medical, Stavanger, Norway) for BLS, a defibrillation trainer (CU Medical Systems, Wonju, Korea) for manual defibrillation, and an Airway Trainer (Laerdal Medical) for tracheal intubation.

The checklist consisted of 20 binary items (performed or not performed), including 7 items for BLS, 7 items for tracheal intubations, and 6 items for manual defibrillation, and a global rating of the overall quality of performance on a 5-point Likert scale (excellent, good, fair, poor, and very poor) (Table 1). This was treated as a numerical scale in the statistical analysis. We sought to ensure the validity of the items by referring to the existing checklist of the Advanced Cardiovascular Life Support provider certification [9].

#### Reliability test of the measurement tool

The Cronbach’s α of the checklist was 0.762.

#### Implementation of peer assessment

In the orientation to the course, students were notified that they would participate as PAs in the resuscitation skills examination. Peer assessment was conducted as follows: the first examinee of the group was assessed only by the FA. After completing the examination, he/she received feedback on his/her performance at the scene. Another faculty member gave the first examinee PA training for 15 minutes. The first examinee then participated in the second examinee’s assessment with the FA. The first examinee’s performance was videotaped, and the last examinee of the group used this video for peer assessment (Fig. 1B). Both student feedback and checklist training were conducted individually without other students attending. To ensure the objectivity of students’ evaluations, they were not informed of whether the peer assessment scores would be included in the final grade. However, it was ultimately not included.

#### Bias

No bias was found in the study scheme.

#### Study size

A post hoc analysis for the paired t-test of means, given an α probability of 0.05, an effect size (d_z_) of 0.5, and a sample size of 54, showed a power (1-β error probability) of 0.947 with 43 degrees of freedom.

### Statistical methods

We used the paired t-test to compare scores between the FA and PAs and used the intraclass correlation coefficient (ICC) to analyze interrater reliability. ICC estimates and their 95% confidence intervals (CIs) were calculated using IBM SPSS ver. 25.0 (IBM Corp., Armonk, NY, USA) based on a 1-way random effects model because each subject was rated by a different set of raters (44 different FA-PA pairs). Values less than 0.5 are indicative of poor reliability, values between 0.5 and 0.75 indicate moderate reliability, values between 0.75 and 0.9 indicate good reliability, and values greater than 0.90 indicate excellent reliability [10]. Bland-Altman plots were used to graphically evaluate the agreement of FA and PA scores by plotting the differences between the FA and PA scores against the FA scores using MedCalc ver. 19.6 (MedCalc Software Ltd., Ostend, Belgium). We used Gwet’s first-order agreement coefficient (AC1) to assess the agreement of individual checklist items between FA and PAs using R ver. 4.0.3 (R Foundation for Statistical Computing, Vienna, Austria; 2020) with the ‘rel’ package version 1.4.2 [11]. In the data processing, if either the FA or PA scores was missing, the other was also considered to be missing. A P-value of <0.05 was considered to indicate statistical significance. The R code is available in Supplement 1.

## Results

**Fig. 2.**
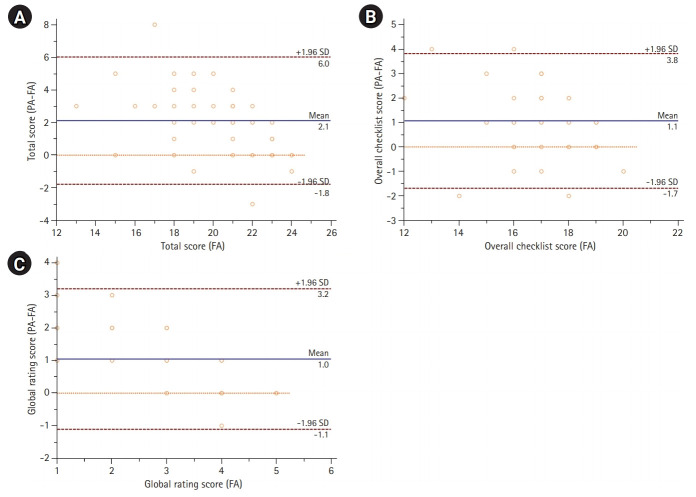
The agreement of faculty assessor (FA) and peer assessor (PA) scores shown by plotting the differences between the FA and PA scores against the FA scores. (A) The agreement of the overall score was determined by plotting the differences between the FA and PA scores against the FA scores. (B) The agreement of the checklist score was determined by plotting the differences between the FA and PA scores against the FA scores. (C) The agreement of the global rating score was determined by plotting the differences between the FA and PA scores against the FA scores. SD, standard deviation.

We analyzed the scores of 44 FA-PA pairs, excluding 10 students who participated in the peer assessment by video. The PA scores were higher than the FA scores (FA versus PAs, mean±standard deviation; 20.2±2.5 versus 22.3±2.4, P<0.001). This was consistent in both the checklist scale (17.2±1.6 versus 18.2±1.8, P<0.005) and the global rating (3.0±1.2 versus 4.0±0.8, P<0.001). Bland-Altman analysis revealed that the 95% limits of agreement were -1.8 to 6.0 points. The limits of agreement exceeded the practically acceptable range (Fig. 2A). Raw data and the analyzed data were available from Dataset 1 and Dataset 2, respectively.

### Agreement for the overall checklist, BLS, tracheal intubation, and manual defibrillation

The agreement for the overall checklist items was poor to moderate (ICC, 0.55; 95% CI, 0.31 to 0.73; P<0.01). Likewise, poor to moderate agreement was also found for BLS, tracheal intubation, and manual defibrillation (Table 2, Dataset 2). The Bland-Altman plot revealed that the 95% limits of agreement were -1.7 to 6.0 points (Fig. 2B).

### Agreement for the global rating of proficiency

The agreement for the global rating was poor (ICC, 0.10; 95% CI, -0.20 to 0.38; P<0.25). The Bland-Altman plot revealed that the 95% limits of agreement were -1.1 to 3.2 points, and the PA score was lower than the FA score in only 1 case (Fig. 2C).

### Agreement for individual checklist items

**Fig. 3.**
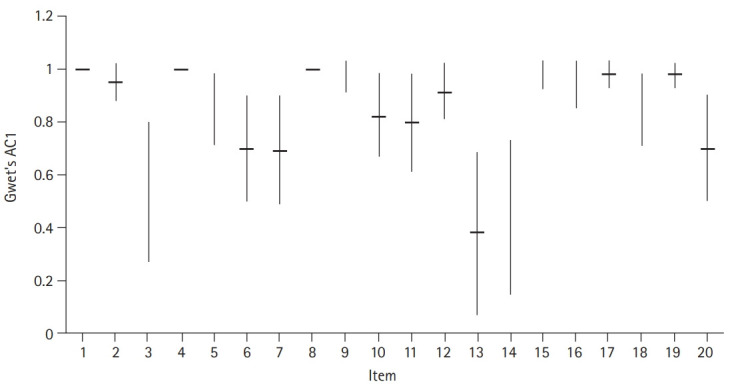
The agreement between individual items between faculty assessor and peer assessors using Gwet’s AC1. The highest agreement was for item 1 (checking responsiveness), item 4 (starting chest compressions immediately after checking the pulse), and item 8 (selecting the appropriate size of a laryngoscope blade for an adult), and the poorest agreement was for checklist items 13 (ventilating the patient at an appropriate rate using bag-mask ventilation) and 14 (ventilating with an appropriate tidal volume). Horizontal lines indicate Gwet’s AC1 values; vertical lines extend to the corresponding 95% confidence interval. Gwet’s AC1, Gwet’s first-order agreement coefficient.

The agreement was highest for checklist item 1 (checking responsiveness), item 4 (starting chest compressions immediately after checking the pulse), and item 8 (selecting the appropriate size of a laryngoscope blade for an adult) with a Gwet’s AC1 of 1.0. However, the agreement was poor for checklist items 13 (ventilating the patient at an appropriate rate using bag-mask ventilation) and 14 (ventilating with an appropriate tidal volume) (Table 1, Fig. 3).

## Discussion

### Key results

Students gave significantly higher scores than the FA on both the checklist scale and global rating. The overall agreement of the assessments by medical students was poor to moderate, and the ventilation-related items had the poorest agreement.

### Interpretation

Students seemed to acknowledge their peers’ partially performed skills as performed skills on the binary checklist consisting only of “performed (yes)” or “not performed (no).” In contrast, the faculty member seemed to have strict standards for partially performed skills. The items related to quantitative parameters such as rates and amounts had poor agreement, such as checking the pulse for 5–10 seconds, a compression rate of 100–120 per minute, compression depth between 5 and 6 cm, a ventilation rate of 10 per minute, and 1/3–1/2 the total volume of the ventilation bag. The global ratings of proficiency given by the PAs were not reliable at all. Proficiency implies a thorough competence derived from training and practice. The criteria of proficiency assessment require a qualitative judgments and could be more subjective than quantitative judgments. Students seem to have been generous with their qualitative judgments of the global rating. The poor agreement on some BLS- and ventilation-related items is considered to have been a technical problem caused by the absence of quantitative measuring equipment.

### Comparison with previous studies

Most peer assessment studies in OSCEs involving resuscitation skills were limited to BLS without tracheal intubation and manual defibrillation [5]. In a study of 9 peer/faculty pairs assessing the BLS of 162 medical students, interobserver agreement was high (>95%). Unlike our study, students who participated as PAs in that study attended a 1-day course given by a European Resuscitation Council BLS instructor and worked with experienced faculty as BLS assessors for 4 hours to consolidate their assessment skills. This implies that PAs should be trained, and that at least 1 day of training is required to train a reliable PA for resuscitation skills assessments. Additionally, the use of devices that objectively measure compression depth and rate, ventilation volume, and similar parameters may improve agreement between FAs and PAs.

According to a review by Khan et al. [12], PAs provided higher global rating scores with moderate to high agreement, variable checklist scores depending on the station, and valuable feedback compared with FAs. The finding that PAs assigned higher global rating scores is consistent with this study, while the finding regarding agreement was not. The 3 main contributions to the reliability of peer assessment are the observed number of relevant performances, the number of peers involved, and the number of competencies being evaluated. Increasing the number of evaluators has a smaller impact on reliability than increasing the number of observations [13]. The poor agreement of global ratings in this study seems to be an issue relating to the aspects of competence being evaluated, rather than a problem of assessor training.

Finn and Garner [14] outlined tips for implementing successful peer assessments, which include considering the relevance of the curriculum, reflective learning, support, and resource allocation in planning. The implementation of peer assessment appears to require a more comprehensive approach, as well as assessor training.

### Limitations

There are several limitations to this study. First, the encounters observed by assessors were not sufficient. Due to coronavirus disease 2019-related social distancing measures, students were not allowed to concentrate in a small OSCE station as PAs. Thus, each student had only the opportunity to observe and assess 1 other student. This is thought to be a major cause of the poor observed agreement. Second, we did not investigate the impact of peer assessment on learning in OSCEs. One of the advantages of peer assessment is learning from peers and the assessment process. This requires students to carefully examine their peers’ performance according to specific criteria. This activity also helps them diversify their approaches and strategies in learning and understanding high- or lower-quality performance. Therefore, students can better understand their learning through insights into performance quality [15]. However, this study did not explore the effects of peer assessment on learning outcomes in depth. The impact of peer assessment on students’ learning will require long-term, qualitative follow-up. In principle, students who show a high level of agreement with an FA may evaluate on behalf of the FA. However, the present study did not analyze this possibility, and further research is needed.

### Conclusion

Senior medical students showed unreliable agreement in peer assessments of advanced resuscitation skills compared to the faculty member’s assessments. Students were more generous in global ratings and showed variable scores on the checklist scale compared with faculty assessments. If a peer assessment is planned in skills education, comprehensive preparation and sufficient assessor training should be carried out in advance.

## Figures and Tables

**Table 1.** The checklist items and agreement of individual items between the faculty and peer assessments

Checklist item	Gwet’s AC1 (95% CI)
Basic life support	
1. Checked responsiveness	1.00 (1.00–1.00)
2. Called for help and defibrillator	0.95 (0.88–1.02)
3. Checked pulse and respiration for 5–10 seconds	0.54 (0.27–0.80)
4. Started chest compression immediately after checking the pulse	1.00 (1.00–1.00)
5. The point of chest compressions was appropriate	0.84 (0.71–0.98)
6. Compression rate was between 100–120 per minute	0.70 (0.50–0.90)
7. Compression depth was between 5 and 6 cm	0.69 (0.49–0.90)
Tracheal intubation	
8. Laryngoscope blade #3 or #4 was used	1.00 (1.00–1.00)
9. Incisor teeth were not injured by laryngoscopy	0.97 (0.91–1.03)
10. Tube was inserted into 21–25 cm at incisor level	0.82 (0.67–0.98)
11. Secured the tube with tape	0.80 (0.61–0.98)
12. Auscultated both the lung and epigastrium after intubation	0.91 (0.81–1.02)
13. Ventilated the patient at an appropriate rate, 30:2 before intubation and 1 ventilation every 6 seconds after intubation	0.38 (0.07–0.68)
14. Ventilated with an appropriate tidal volume, approximately 1/3-1/2 of the total bag volume	0.44 (0.15–0.73)
Defibrillation	
15. Decision to shock was appropriate at the first rhythm	0.97 (0.92–1.03)
16. Decision to shock was appropriate at the second rhythm	0.94 (0.85–1.03)
17. Energy level was appropriate (200 J)	0.98 (0.93–1.03)
18. Locations of the defibrillation paddles were appropriate	0.84 (0.71–0.98)
19. Shouted to clear everyone just before defibrillation	0.98 (0.93–1.02)
20. Resumed chest compression immediately after defibrillation	0.70 (0.50–0.90)

Gwet’s AC1, Gwet’s first-order agreement coefficient; CI, confidence interval; ICC, intraclass correlation coefficient, Value* is presented as ICC(95% CI).

**Table 2.** The ICC of overall checklist items, BLS, tracheal intubation, and manual defibrillation

Variable	Measure	ICC (95% CI)	F-test with true value 0			
			Value	df1	df2	Significance
Overall checklist	Single measures	0.55 (0.31 to 0.73)	3.46	43.00	44.00	<0.0001
BLS	Single measures	0.19 (-0.11 to 0.46)	1.47	43.00	44.00	0.1044
Tracheal intubation	Single measures	0.51 (0.26 to 0.70)	3.11	43.00	44.00	0.0001
Manual defibrillation	Single measures	0.49 (0.23 to 0.68)	2.91	43.00	44.00	0.0003

Gwet’s AC1, Gwet’s first-order agreement coefficient; CI, confidence interval.

## Footnotes

**Data availability**
Data files are available from Harvard Dataverse: https://doi.org/10.7910/DVN/SABNDD Dataset 1. Raw score data by professors and students (peers) for 20 item measurement tools for the advanced resuscitation skills examination.

Dataset 2. Scores according to each category and overall scores by professors and students (peers) for 20 item measurement tools for the advanced resuscitation skills examination.

## Supplementary materials

Supplementary files are available from Harvard Dataverse: https://doi.org/10.7910/DVN/SABNDD

Supplement 1. R code using the ‘rel’ package for the Gwet’s first-order agreement coefficient between the faculty member’s assessment and peer assessments.

Supplement 2. Audio recording of the abstract.
