# Supplementary material for: Agreement between medical students’ peer assessments and faculty assessments in advanced resuscitation skills examinations in South Korea
Source: J Educ Eval Health Prof. 2021 Mar 25;18:4. doi: 10.3352/jeehp.2021.18.4 (PMC8089466; doi:10.3352/jeehp.2021.18.4)
Supplement: Supplementary file 3 — Supplement 1. R code using the ‘rel’ package for the Gwet’s first-order agreement coefficient between the faculty member’s assessment and peer assessments. [file jeehp-18-04-suppl.docx]

library(tidyverse)

library(readxl)

library(rel)

SCORECARD <- read_excel("binary_checklist.xlsx",na="-")

SCORECARD <- transmute_all(SCORECARD,as.integer)

RESULT <- NULL

for (i in 1:20) {

SCORE <- select(SCORECARD,starts_with(paste(i,"_",sep="")))

gwet <- gac(data=SCORE, kat=2, weight="unweighted", conf.level=0.95)

RESULT <- bind_rows(RESULT, c(i, gwet$est, gwet$lb, gwet$ub))}

colnames(RESULT) <- c("ITEM","GWET_AC1","CI95_LOW","CI95_HIGH")

write_csv(RESULT,"dicotomous.csv")
